# Tensile Strength and Failure Types of Direct and Indirect Resin Composite Copings for Perio-Overdentures Luted Using Different Adhesive Cementation Modalities

**DOI:** 10.3390/ma13163517

**Published:** 2020-08-10

**Authors:** Raffaele Cesca, Vera Colombo, Bruna Ernst, Luigi Maria Gallo, Mutlu Özcan

**Affiliations:** 1 Laboratory of Physiology and Biomechanics of the Masticatory System, Clinic for Masticatory Disorders, Center for Dental and Oral Medicine, University of Zurich, 8032 Zurich, Switzerland; raffaele.cesca@libero.it (R.C.); Vera.Colombo@zzm.uzh.ch (V.C.); Luigi.Gallo@zzm.uzh.ch (L.M.G.); 2 Clinic for Reconstructive Dentistry, Division of Dental Biomaterials, Center for Dental and Oral Medicine, University of Zürich, 8032 Zurich, Switzerland; Bruna.Ernst@zzm.uzh.ch

**Keywords:** adhesion, coping, retention elements, tensile strength

## Abstract

Perio-overdenture design helps to reduce periodontal diseases and secondary caries on abutment teeth. Composite copings can be cemented adhesively to the abutment teeth with different techniques. In this study, direct/indirect resin composite copings for perio-overdentures, luted using different adhesive cementation modalities were compared. Human teeth (N = 40) were prepared to receive spherical attachment copings and randomly divided into four groups: (1) resin-composite copings bonded directly (DC), (2) composite copings made indirectly, luted with dual-polymerized resin cement (ICV), (3) composite copings made indirectly, bonded with resin composite (ICT), (4) composite copings made indirectly, bonded with resin composite after the immediate dentin sealing method (IDS). Specimens were tested for tensile failure and one-way ANOVA (alpha = 0.05) was performed and the two-parameter Weibull modulus, scale (*m*) and shape (_0_) were calculated. Mean tensile load (N) was significantly higher for Group IDS (238 ± 81) than for the other groups (144 ± 53–184 ± 46) (*p* < 0.05). Group IDS (0.54 ± 0.25 mm) showed significantly higher deformation (mm) than other groups (0.2 ± 0.1–0.32 ± 0.15) (*p* < 0.05). Weibull distribution presented lower shape (_0_) for DC (3.33) compared to other groups (3.57–4.99). Cohesive coping failures were more frequent in Group IDS (60%) and mixed failures in other groups (40–60%). In conclusion, IDS copings could be preferred over other fabrication and adhesion modalities.

## 1. Introduction

In order to overcome some of the problems encountered with conventional overdentures, such as plaque accumulation on abutment teeth, inflammation of the periodontium, trauma on the supporting mucosa, elevated caries risk and patient acceptance, an alternative concept of framework has been proposed for the hybrid prostheses in the early eighties [1,2]. Originally, perio-overdentures were anchored to the abutment teeth by means of precision attachments soldered to golden copings [3,4]. This technique relies on mechanical retention. With the advances in adhesive technologies, such as chemical, physical or physico-mechanical surface conditioning methods, the use of resin composite copings has been introduced [5,6]. Resin composite copings can either be directly molded and bonded to intraradicular dentin (direct technique) or prepared in the laboratory and then adhesively cemented to the abutment teeth (indirect technique) [7]. This approach, which employs adhesive procedures, eliminates the need for additional impressions. Moreover, the possibilities of repairing a tooth restored with resin composite [8,9] would allow for maintaining the coping in situ compared to those with gold ones.

Two different kinds of retention elements are currently available on the market for resin composite copings, namely, those with root post and the ones without post. Although in the past two decades, the use of fiber posts has increased, the standard choice of material for the posts remains as metal [10]. The fit of the metal posts as copings relies on retention through friction in the root canal and in case of a fracture of the metal, an unfavorable fracture pattern may be detrimental to the longevity of the tooth yielding to extraction [11,12].

The current restorative trends in dentistry aim at preserving as much dental tissue as possible. The possibility of restoring teeth with resin composite copings without the use of root posts would fulfil this requirement where scarce clinical data are available [7]. However, the durable adhesion to dental tissues could only dictate the longevity of such treatment options in partially edentulous patients. To date, no study has analyzed the adhesive behavior of resin composite copings without posts cemented using either resin composite or luting cements. Thus, information for a sound clinical protocol is lacking on the adhesion of such copings without post, providing that such copings undergo tensile and/or compressive loading when adhesively cemented to the intraradicular dentin. The success of perio-overdentures would also decrease the number of implant applications and the related possible infection incidence around the implants.

The objectives of this study, therefore, were to compare the tensile strength of resin composite copings fabricated directly or indirectly for perio-overdentures luted in the root canal using different adhesive cementation modalities. The null hypothesis tested was that adhesive cementation protocols used for direct and indirect copings would not significantly affect the tensile strength.

## 2. Material and Methods

### 2.1. Specimen Preparation

Extracted human teeth (maxillary central incisors, canines and premolars) (N = 40), due to periodontal reasons, were kept in distilled water until the experiments at 4 °C and during experiments at 23 °C. The reasons for the extraction of the teeth used in this study were unrelated to the study. Written informed consent for the research purpose of the extracted teeth was obtained by all donors prior to extraction according to the directives set by the National Federal Council. Ethical guidelines were strictly followed, and irreversible anonymization was performed in accordance with State and Federal Law [13,14,15]. Under water cooling, the coronal sections of the teeth were removed 1 mm below the cement-enamel junction (CEJ) using a diamond bur (Type 859, Intensiv SA, Lugano, Switzerland). After removal of the pulp, intraradicular canals were cleaned. The root canals were not filled in order to avoid possible contamination of the intraradicular dentin with the subsequent root canal filling materials.

**Figure 1:**
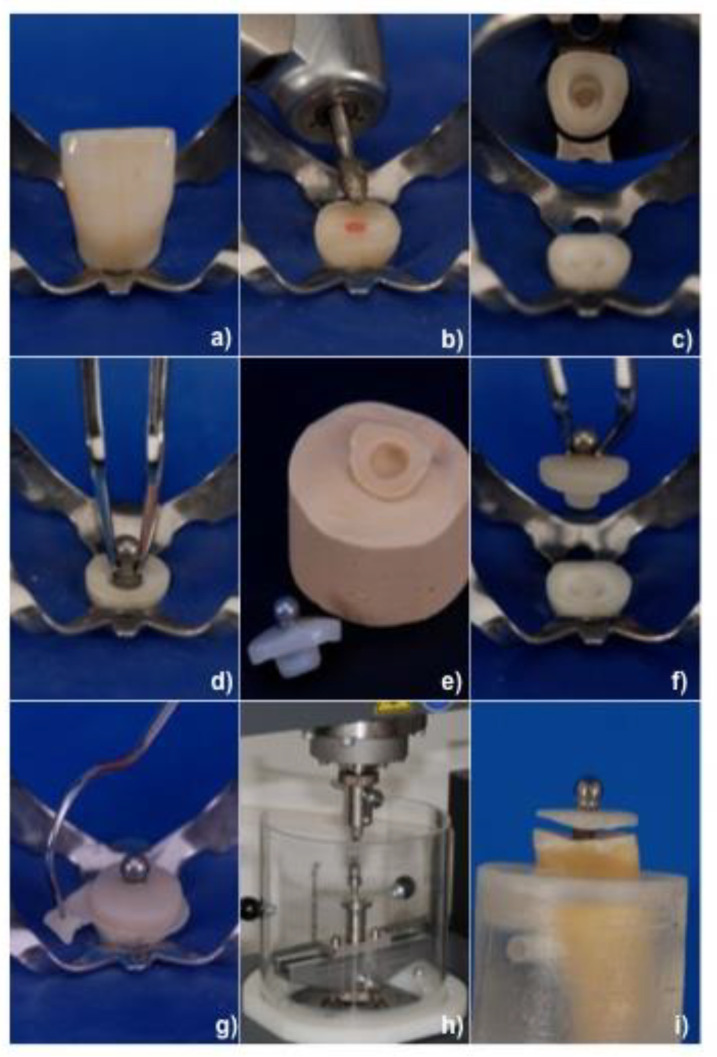
(**a**–**i**) Sequential phases of specimen preparation and testing. (**a**) Extracted tooth isolated with rubber dam to increase the working conditions of the operator, (**b**) removal of coronal part of the tooth with diamond bur at cement-enamel junction (CEJ), preparation of occlusal/incisal surface of tooth using diamond bur with light concavity in order to increase adhesive surface, (**c**) preparation of elliptical inlay to achieve stability and avoid rotational movement during cementing procedure, (**d**) checking vertical position of retentive male part (GP-Ball), (**e**) obtaining stone cast from silicon impression of the preparation and molding coping at the laboratory, (**f**) try-in of the coping, (**g**) cementation of the coping on the tooth, (**h**) positioning the specimen in the Universal Testing Device, (**i**) tensile test until failure of the coping.

All specimens were prepared in order to receive resin composite copings with spherical attachments (GP-Ball without post, Kaladent AG, Unor Labor Service, Zurich, Switzerland) (Figure 1a–i). The teeth (n = 10 per group) were randomly divided into 4 groups and in each group resin composite copings were bonded to the teeth with different adhesive cementation modalities (Table 1).

Group DC: The teeth were restored with direct resin composite (Tetric Ceram, Ivoclar Vivadent, Schaan, Liechtenstein) copings using a multi-step etch-and-rinse adhesive system (Syntac System, Ivoclar Vivadent). Dentin surface was conditioned with phosphoric acid for 15 s and rinsed with water. Primer, adhesive and bonding agent (Syntac System, Ivoclar Vivadent) were subsequently applied according to the manufacturer’s instructions and photopolymerized (Bluephase C8 LED, Ivoclar Vivadent; light output: 1200 mw/cm^2^) for 40 s. The resin composite copings were molded incrementally with the aimed increment thickness of 2 mm on the teeth and the spherical attachments (GP-Ball without post, Kaladent AG, Unor Labor Service, Zurich, Switzerland) were embedded in the resin composite mass. Initially, each increment of resin composite layer was photopolymerized for 20 s and the copings were then additionally polymerized for 60 s.

Group ICV: The teeth in this group were restored with indirectly fabricated resin composite copings (Tetric Ceram, Ivoclar Vivadent) cemented with dual polymerizing luting cement (Variolink II, Ivoclar Vivadent). The resin composite was indicated for direct applications, but it was processed in an indirect fashion. Impressions of the prepared teeth were made with polyvinylsiloxane impression material (Provil Novo, Heraeus Kulzer GmbH, Hanau, Germany) and stone cast (Elite model type 3, Zhermack, Rovigo, Italy) were obtained. Resin composite copings were built up in the laboratory (Tetric Ceram) and photopolymerized in an oven (Dentacolor XS Kulzer, Heraeus Laborgeräte AG, Hanau, Germany) for 90 s. The cementation surfaces were then airborne-particle abraded (Airsonic Minisandblaster, Hager and Werken, Duisburg, Germany) (50 µm Al_2_O_3_, 10 s, 2 bar pressure; distance: 10 mm). The teeth were provisionally restored with a restoration material (Cavit, 3M ESPE, St. Paul, MN, USA).

After one week, the temporary restoration material was removed, copings were ultrasonically cleaned in distilled water (Telsonic Power Cleaning, Telsonic AG, Bronschhofen, Switzerland), the tooth surface was airborne-particle abraded with 50 µm Al_2_O_3_ particles and the adhesive system (Syntac System, Ivoclar Vivadent) was applied according to the manufacturer’s instructions and photopolymerized for 40 s. Resin composite copings were rinsed with ethanol, and silanized with one coat of 3-(Methacryloyloxy)propyltrimethoxysilane (MPS) silane coupling agent (Monobond S, Ivoclar Vivadent). After waiting for the reaction of silane for 60 s, one coat of bonding agent (Heliobond, Ivoclar Vivadent) was applied, air-thinned and photopolymerized for 20 s. The indirect copings were then cemented using dual polymerized luting cement (Variolink II, Ivoclar Vivadent). The copings were photopolymerized for 40 s from four directions and additionally polymerized for 20 s from each direction after the application of glycerin gel.

Group ICT: In this group, the teeth were restored with an indirectly fabricated resin composite (Tetric Ceram, Ivoclar Vivadent) as described in Group II but bonded with pre-heated resin composite (Tetric Ceram, Ivoclar Vivadent) using the same adhesive system (Syntac System, Ivoclar Vivadent). The copings were then photopolymerized for 40 s from four directions and additionally polymerized for 20 s from each direction after the application of glycerin gel.

Group IDS: The teeth in this group were restored with indirectly fabricated resin composite copings (Tetric Ceram, Ivoclar Vivadent) as described in Group II and bonded with pre-heated resin composite (Tetric Ceram, Ivoclar Vivadent) using the same adhesive system (Syntac System, Ivoclar Vivadent). This group received immediate dentin sealing prior to impression making. All prepared teeth were conditioned again using the same adhesive system. The copings were bonded and photo-polymerized for 40 s from four directions and additionally polymerized for 20 s from each direction after the application of glycerin gel. In the direct group, only the surface of the coping was not polymerized because it is inhibited by oxygen while the interface, being covered by the other increments, was completely polymerized. In the indirect group, on the other hand, the peripheral composite/cement margin between tooth and coping remained in contact with the air and therefore its complete polymerization is inhibited.

In Groups ICV and IDS, no air-abrasion was practiced prior to impression making but before adhesive application in order to remove possible contaminants. Air-abrasion was achieved using 30 µm alumina particles using a chairside air-abrasion device (Dento-Prep, RØNVIG A/S, Daugaard, Denmark; pressure: 2.5 bar, distance: 10 mm, duration: 5 s in circling motion) [16,17].

Specimens of all groups were embedded in cylindrical resin mass (Paladur, Heraeus Kulzer GmbH) until the CEJ and kept for 1 week in distilled water prior to the mechanical tests.

### 2.2. Calculation of Adhesive Interface

**Figure 2:**
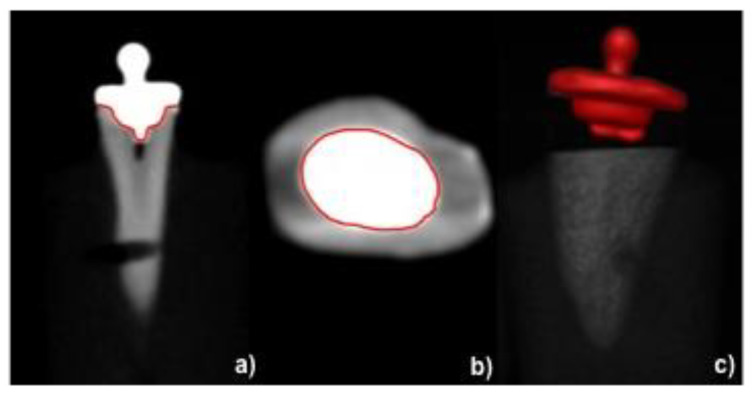
(**a**–**c**) Digital volume tomography (DVT) image of restored tooth on (**a**) sagittal, (**b**) coronal section, and (**c**) the perspective view of 3D reconstructed surface. Contours of cemented surface, highlighted in red, are determined for each DVT slice and surface reconstructed to calculate the adhesive area.

**Figure 3:**
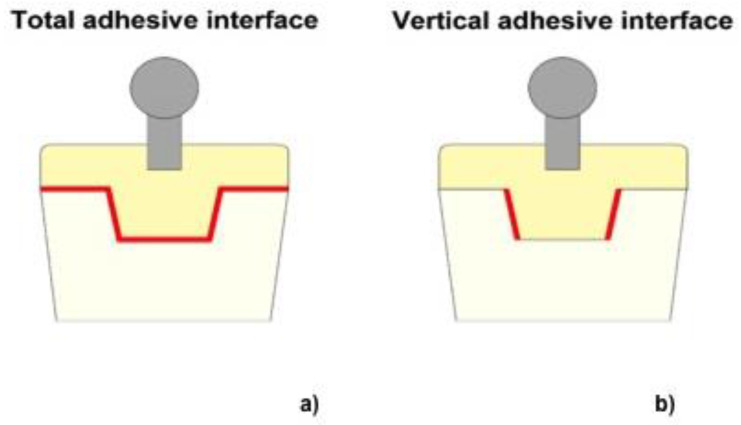
Schematic of definition of (**a**) total and (**b**) vertical adhesive interface considered during adhesive area calculation.

Digital volume tomography (DVT) (KaVo 3D Exam, KaVo Dental GmbH, Biberach, Germany) images were made for each tooth with a voxel size of 0.25 mm × 0.25 mm × 0.25 mm (Figure 2a–c). The goal of the DVT was to determine the shape of the interface between the materials and to calculate the surface on which the tensile strength is applied. Afterwards, the obtained images were analyzed using 3D image analysis software (AMIRA, FEI VSG, Berlin, Germany) in order to measure the adhesive interface between the resin composite copings and the prepared dentin surface. Thus, from the overall preparation, in the data analysis horizontal surfaces were neglected and only the vertical adhesive interfacial area was considered for stress calculation (Figure 3a,b).

### 2.3. Mechanical Tests and Failure Type Analysis

The specimens were tested for tensile strength in a Universal Testing Machine (Zwick/Roell Z010, Zwick GmbH, Ulm, Germany). A custom-made jig was constructed in order to mount the specimens in the device in a reproducible way. Each specimen was then pulled from the attachment ball until copings fractured or debonded (1 mm/min). Tensile bond and deformation levels to failure were then calculated.

All copings’ surfaces were further analyzed and failure types were scored as follows: A: Adhesive, coping debonded adhesively from the tooth with no remnants of cement/adhesive resin on the tooth surface; M: Mixed, partial remnants of cement/adhesive resin on the coping; C: Cohesive, coping material remained completely on the tooth surface.

### 2.4. Statistical Analysis

Considering the mean values obtained with a difference of approximately 2 MPa between each group and the highest standard deviation of 3.6 in adhesion data, a sample size of 10 in each group was calculated (IBM SPSS Software V.19 for Windows and G*Power, Chicago, IL, USA) to have more than 80% power to detect a difference in means of tensile bond strength using a two-group Satterthwaite t-test (IBM SPSS Software V.19 for Windows, Chicago, IL, USA) (alpha = 0.05). The obtained data were analyzed statistically (SPSS Software V.20, Chicago, IL, USA). Normal distribution was tested using Kolmogorov–Smirnov and Shapiro–Wilk tests. Normality was not violated and therefore univariate analysis of variance (1-way ANOVA), Tukey’s and Tamhane post-hoc tests for the adhesion surfaces, effects of adhesion methods on deformations and stress values were performed. Furthermore, 2-parameter Weibull distribution was used for calculating Weibull modulus, scale (*m*) and shape (_0_) in order to assess the reliability of adhesion (Minitab Software V.16, State College, PA, USA). In all tests, *p* < 0.05 was considered to be statistically significant.

## 3. Results

The mean total adhesive interfaces between dentin and composite resin, determined by a 3D software program, were 49.7 ± 8 mm^2^, 51.1 ± 8.1 mm^2^, 49.4 ± 7.1 mm^2^, 54 ± 8.4 mm^2^, for DC, ICV, ICT and IDS, respectively, being not statistically significant (*p* > 0.05). The mean values for the vertical adhesive interfaces were 26.6 ± 5.1 mm^2^, 24.9 ± 5.4 mm^2^, 21.9 ± 5.4 mm^2^, 25.5 ± 5.6 mm^2^, for DC, ICV, ICT and IDS, respectively, being also not statistically significant (*p* > 0.05).

Mean tensile strength (N) and adhesion (MPa) were significantly higher for Group IDS (238 ± 81 N; 12.5 ± 3.3 MPa) compared to those of other groups (144 ± 53–184 ± 46 N; 5.6 ± 2.2–9.5 ± 2.8 MPa) (*p* < 0.05) (Table 2). This finding was supported by the significantly higher deformation level (mm) of Group IDS (0.54 ± 0.25 mm) versus the others (*p* < 0.001).

Ultimate mean tensile bond strength (MPa), obtained from the forces acting on the vertical adhesive surface were 5.6 ± 2.2, 7.4 ± 3.6, 9.5 ± 2.8, 12.5 ± 3.31 for DC, ICV, ICT, and IDS, respectively. Statistical differences were found between DC and IDS (*p* = 0.041).

Weibull distribution presented lower shape (_0_) for DC (3.33) compared to other groups (3.57, 3.6, 4.99, respectively).

The incidence of cohesive coping failures (Type C) within the coping material was more frequent in Group DC (50%) and in Group IDS (60%) followed by mixed (Type M) (40%) failures showing more reliable adhesion while in other groups mainly mixed (Type M) (50, 40, 60% for DC, ICV, ICT, respectively) and adhesive (Type A) (0, 40, 20% for DC, ICV, ICT, respectively) failures were observed.

## 4. Discussion

High and therefore unfavorable C-factor for the root canal contributes maximum polymerization stress of luting cements in the root canal [18,19]. In that respect, omitting the use of a root post in copping fabrication would be a favorable approach. Yet, durability of such a therapy using composite copings would rely mainly on the adhesion of the copping in the coronal portion of the canal. This study was undertaken in order to compare the tensile strength of directly and indirectly fabricated resin composite copings for perio-overdentures luted in the root canal using different adhesive cementation modalities. Based on the results of this study, since mean tensile strength and thereby adhesion values were significantly affected by adhesion modalities in conjunction with coping fabrication methods, the null hypothesis could be rejected.

Bond strength tests are the most frequently used test methods to screen the performance of adhesives. However, typically specimens with standard geometries are used in such studies [20,21,22]. Investigating the retention of posts or copings in the root canal opening or in the root could be considered more challenging due to the heterogenous geometry and the biological nature of the root canal. Nevertheless, in this study, in an attempt to measure the bond strength, the bonded surface area was measured using 3D image analysis. The adhesive properties of the cement are different when the force is applied perpendicularly to the adhesive surface or parallel [23]. Due to the complex geometry of the preparation, which is not a flat surface, the vertical and horizontal areas might have a different influence on the tensile strength. Furthermore, it is known that dentinal tubuli in the root canal run perpendicularly to the root surface [24], allowing the penetration of the filling material into the dentinal tubuli [25]. This might influence the tensile behavior of the coping. For these reasons, vertical interfacial area was considered for the stress analysis. Nevertheless, both the tensile strength and tensile bond strength were significantly higher in group IDS where resin composite was fabricated indirectly compared to other groups and more distinctly to the direct fabrication method (DC).

Fabrication of resin composites in a laboratory processing method is achieved using a polymerization device where the resin is processed both with light and heat yielding to improve the degree of conversion of the resin compared to the direct method [26,27]. Although an incremental technique was employed and the resin composite was initially pre-heated in order to decrease its viscosity and increase polymerization kinetics [28,29], the lowest results obtained in the DC groups could be partially attributed to less wettability of the resin composite to the dentin surface. In fact, DC method without requiring any additional surface conditioning steps and the use of luting cement would have decreased the clinical workflow significantly. However, even the same resin composite was used in ICT and IDS groups, and higher tensile forces were needed to debond the coping. This finding clearly indicates that not only the adhesion of the resin materials to the root dentin but also the cohesive strength of the coping material affects the forces required to debond the coping. It also has to be noted that both the resin composite and the resin cement were methacrylate-based resin materials and all copings were cemented to the coronal portion of the root where improved adhesion has been reported compared to those of middle and apical parts [30,31,32]. Since higher results were obtained with all indirect methods (ICV, ICT, IDS), compared to direct method (DC), indirect coping fabrication method could be considered to provide more favorable mechanical properties, cemented either using resin composite or dual-polymerized resin cement. Likewise, Weilbull modulus was the lowest with the DC method demonstrating less reliable adhesion. The results of this study were comparable with those of Perez et al., where a 7 to 8 MPa bond strength was reported [30]. On the other hand, the results were higher than those of Sukuroglu et al. [31] where adhesion results ranged between 3.8 and 5.6 MPa in the coronal region of the root. Both of these studies [30,31] reported push-out bond strength results whereas in this study tensile bond strength was measured. Nevertheless, the use of dual polymerized cement in this study resulted in higher bond strength values with self-adhesive cements [30] in the coronal parts of the intraradicular dentin.

In this study, immediate dentin sealing was achieved using the same adhesive resin system based on the etch-and rinse technique that is still considered the gold standard in adhesion to dentin [33,34,35]. This type of sealing method, prior to impression making, eliminates the possible contamination of the root dentin with the silicone-based impression material. Inoue et al. [36] compared the bonding capacity of self-etch adhesives to mid-coronal dentin to “total-etch” adhesives. In that study, one-step “self-etch” adhesives presented the lowest bond strength values but were not significant compared to those of two-step “self-etch” adhesives. Moreover, good bonding to root canal dentin, despite a thin hybrid layer, was achieved, which was about 1 µm thick. Therefore, a three-step “total-etch” adhesive which was chosen as the adhesive system in this study. The use of the “total-etch” adhesive system provides lower pH results in higher results compared to self-adhesive cements, which do not necessitate the use of acid etching due to the acidic monomer in their composition [34,35,37].

The tensile strength and tensile bond results of this study should be coupled with the failure analysis that was made on both the coping and the retainer/attachment surface. The high number of cohesive failures in the IDS group implies that the adhesion of the resin composite to the tooth and the retainer exceeded that of the cohesive strength of the coping, which also correlated with the high mean tensile force and adhesion results presented in this group. On the other hand, the high number of adhesive failures indicate less favorable adhesion of the copping. Clinically, such failure type may lead to debonding of the copping from the tooth surface leaving cement on the dentin surface that needs to be removed when recementation is contemplated. This could eventually lead to loss of the precise fit of the retainer. Cohesive failures, on the other hand, presented the situation where coping material remained completely on the tooth surface. Such a failure type is related to the cohesive strength of the coping material. Thus, physico-chemical properties of a resin composite, including mechanical features and degree of polymerization of the resin composite, may affect the failure types in that higher polymerization and could yield to a more brittle material and result in a cohesive failure in the material itself before debonding of the coping from the root substrate.

In this study, the coping surface was only air-abraded using 50 µm Al_2_O_3_ and silanized using an MPS silane. Other physico-chemical surface conditioning methods [16] and the use 10-methacryloxydecyl dihydrogen phosphate (10-MDP) containing silane coupling agents [38,39] may increase the adhesion of resin materials tested to the retainer surface which needs to be further investigated.

The results of this study should additionally be verified after aging conditions. Standard endodontic treatment contaminates the root canal which was not practiced in this study. The reason for exclusion of root canal filling procedures was to evaluate the tensile force and adhesion values without any confounding factors related to contamination of the dentinal tubuli from such root canal filling materials and also to decrease the dehydration of the teeth. This could be considered a limitation of this study and should be further investigated.

## 5. Conclusions

From this study, the following could be concluded:

1. Indirectly fabricated resin composite copings bonded with the same resin composite after immediate dentin sealing of the canal presented the highest retention compared to other coping fabrication and adhesive cementation methods.
2. Ultimate tensile bond values and the Weilbull modulus were the lowest with directly fabricated resin composite coping without the use of adhesive cement, meaning less reliable adhesion.
3. The group cemented after immediate dentin sealing showed higher incidence of cohesive failures in the coping indicating that the adhesion to the root dentin exceeded that of the cohesive strength of the coping material.

## Figures and Tables

**Table 1:** Description of experimental groups with regard to preparation method, luting cement, bonding agent and intraradicular dentin sealing method.

Preparation Method/Materials	Group DC	Group ICV	Group ICT	Group IDS
Coping preparation method	Direct	Indirect	Indirect	Indirect
Luting material	Resin composite (Tetric Ceram)	Dual polymerized resin cement (Variolink II)	Resin composite (Tetric Ceram)	Resin composite (Tetric Ceram)
Adhesive system	Etch-and-rinse adhesive system (Syntac)	Etch-and-rinse adhesive system (Syntac)	Etch-and-rinse adhesive system (Syntac)	Etch-and-rinse adhesive system (Syntac)
Intraradicular dentin sealing	No Immediate Dentin Sealing	No Immediate Dentin Sealing	No Immediate Dentin Sealing	Immediate Dentin Sealing

**Table 2:** Mean ± standard deviation of maximum tensile force, adhesion and deformation levels observed in each experimental group. The same uppercase small letters indicate no significant difference in each row according to 1-way ANOVA, Tukey’s and Tamhane post-hoc tests (*p* < 0.05).

Measured Parameters	Group DC	Group ICV	Group ICT	Group IDS
Tensile Force (N)	144 ± 53 ^a^	167 ± 55 ^a,b^	184 ± 46 ^a,b^	238 ± 81 ^c^
Adhesion (MPa)	5.6 ± 2.2 ^a^	7.4 ± 3.6 ^a,b^	9.5 ± 2.8 ^a,b^	12.5 ± 3.31 ^c^
Deformation level (mm)	0.32 ± 0.15 ^a^	0.29 ± 0.1 ^a,b^	0.2 ± 0.1 ^a,b^	0.54 ± 0.25 ^c^

## Clinical Relevance

Considering the tested materials, for better retention and durability of copings used for perio-overdentures, indirect fabrication method using resin composite cemented with the same resin material after immediate dentin sealing of the canal should be preferred to direct fabrication method.
